# Editorial: The Advances in Semen Evaluation

**DOI:** 10.3389/fvets.2022.874937

**Published:** 2022-03-14

**Authors:** Heriberto Rodriguez-Martinez, Michael Robert McGowan, Szabolcs Nagy, Manuel Alvarez-Rodríguez

**Affiliations:** ^1^Department of Biomedical and Clinical Sciences (BKV), BKH/Obstetrics and Gynaecology, Faculty of Medicine and Health Sciences, Linköping University, Linköping, Sweden; ^2^School of Veterinary Science, The University of Queensland, Gatton, QLD, Australia; ^3^Hungarian University of Agriculture and Life Sciences, Keszthely, Hungary; ^4^Department of Animal Health and Anatomy, Faculty of Veterinary Medicine, Universitat Autònoma de Barcelona, Barcelona, Spain

**Keywords:** semen, fertility, cryopreservation, animal production, OMICS

Although there have been significant advances in our ability to accurately identify infertile and moderate to severely subfertile breeding males, our ability to accurately identify highly fertile males continues to be quite challenging. Apart from motility and kinetic parameter analysis, a number of studies investigating the impact on fertility outcomes of spermatozoa radical oxidative stress (ROS), Total Antioxidant Capacity (TAC), DNA fragmentation, membrane status (acrosome damage, lipid peroxidation, cholesterol influx, apoptosis-like changes), and capacitation-like status have been conducted. However, there is still a lack of a complete definition of the components of semen, not only the spermatozoa itself but also seminal plasma components that affect fertility outcomes. Thus, the development of new analytical tools to accurately define sperm quality is critical.

In the first paper, Anel-López et al. compared the effects on sperm quality and fertility outcomes of use of three commonly used antibiotics in ram semen extenders. Using computer assisted sperm analysis, sperm motility parameters were significantly lower for semen extended with gentamycin. Further, it was shown that there were no significant differences in fertility outcomes between semen extended without an antibiotic vs. semen treated with penicillin-streptomycin, globally the most commonly used extender antibiotic. This finding was consistent with immediate post-thaw results of simultaneous flow cytometric assessment of sperm viability (Caspase 3 and 7 Activity, ROS generation) and prompts the question, “in the absence of venereal pathogens is the antibiotic treatment of semen necessary”. In a larger study examining the relationship between a range of conventional and new post-thaw laboratory measures of ram sperm quality, Mendoza et al. demonstrated that the percentage of intact membrane, non-capacitated (IM-NC) spermatozoa (evaluated using the chlortetracycline assay in combination with ethidium homodimer) in extended chilled semen used for cervical artificial insemination was positively correlated with fertility outcomes.When the analysis was restricted to insemination conducted during the breeding season, an additional three new measures of sperm quality (oxygen consumption, apoptotic-like markers caspase activation) were significantly correlated with fertility outcomes. Modeling indicated that the use of semen with higher percentages of IM-NC and DNA-intact sperm would result in fertility outcomes greater than the population mean.

Three papers examined the impact on sperm quality of direct or indirect administration of plant derived medicinal substances. Sobeh et al. reported the findings of a study investigating the impact on sperm quality of addition of a polyphenol-rich extract, derived from the bark of *Entada abyssinica*, to a standard semen extender. It was concluded that the extracts's potent antioxidant capacity contributed to the observed improvement in sperm progressive motility and plasma membrane integrity. Greco et al. investigated the effect of administration of Tetrahydrocannabinol (THC), maca (a traditional Andean crop), and their combination on testicular tissue and semen parameters in mice. The natural anti-oxidant properties of maca have been shown to protect testicular cell membranes and mitochondria from oxidative stress. Overall, administration of maca, reduced the deleterious effect of THC on testicular parenchyma and semen production. Mao et al. tested the effect of dietary *Echinacea purpurea* (EPE) treatment on the reproductive function of streptozotocin-nicotinamide-induced diabetic rats. EPE treatment significantly increased sperm progressive motility and decreased the percentage of sperm abnormalities. Interestingly, they demonstrated that feeding of EPE increased sperm enzymatic antioxidants (superoxide dismutase, catalase activities, and glutathione), whereas proinflammatory cytokines, such as NO, IL-1b, and TNF-a were decreased.

Three papers examined the impact of chilled and frozen semen storage on sperm quality. Suwimonteerabutr et al. investigated the impact on sperm quality of the addition of butaphosphan and cyanocobalamin to a standard semen extender for chilled boar semen. Overall, supplementation of the semen extender resulted in significant improvements in progressive motility, sperm viability and plasma membrane integrity through to Day 7 of chilled storage. Using a unique approach Wang et al. investigated the molecular mechanisms of sperm cryoinjury and cryoresistance by comparing the piRNA profiles of boar sperm with that of Giant Panda sperm, the latter being known to be quite cryotolerant. They concluded that observed species difference in the profiles involved in the cAMP signaling pathway may be responsible for the difference in cryotolerance of boar and Giant Panda sperm. Continuing in this theme O'Brien et al. investigated whether domestication of ungulates is likely to have altered the sensitivity of their sperm to laboratory manipulation and chilled storage. Using principal components analysis they showed that mitochondrial membrane integrity, oxidative stress level (percentage of low levels of reactive oxidative species) and curvilinear velocity of sperm after chilled storage were the most important biomarkers defining differences between the sperm of domesticated and wild species of pigs, sheep, and goats, respectively.

It is now recognized that instead of semen containing a homogenous population of spermatozoa, it consists of subpopulations of spermatozoa related to period of spermatogenesis and spermatozoal maturation. Gacem et al., using a computer-aided sperm motility analysis system recording sequences at high frequency (250 frames per second), compared the kinematics of sub-populations of spermatozoa from two different horse breeds with that of donkeys. Three distinct sub-populations of motile spermatozoa were defined for both species. The predominant subpopulation consisted of spermatozoa with very fast velocity characteristics and a linear trajectory with a high beat frequency.

In the first of the four reviews, Khan et al. focused on how studies using OMICS technologies (proteomics and transcriptomics) can improve our understanding of the mechanisms of cryo-injury and cryo-tolerance. Systematic application of these technologies could contribute to the optimization of current farm animal cryopreservation protocols. The ultimate goal is the identification of biomarkers that accurately predict spermatozoa freezability. Continuing this focus, Evans et al. examined how lipidomics technologies could be applied to accurately characterize the fatty acid composition and their biological roles in ejaculated, stored, and cryopreserved spermatozoa. Özbek et al. reviewed how OMICS technologies have been and further could be applied to improve identification and selection of more fertile bulls. The final review focused on the critical issue of the level of agreement between laboratories, specifically for the assessment of the percentage of morphologically normal spermatozoa (Perry). A programme to support standardized examination of fixed samples of bull semen by a network of commercial laboratories is described, together with the standardized reporting of specific abnormalities and the maximum percentages of each, based on published reports of their impact on fertility.

## Author Contributions

HR-M, MM, SN, and MA-R contributed to the conceptualization, writing of the original draft, and review and editing. All authors approved the final version of this editorial.

## Conflict of Interest

The authors declare that the research was conducted in the absence of any commercial or financial relationships that could be construed as a potential conflict of interest.

## Publisher's Note

All claims expressed in this article are solely those of the authors and do not necessarily represent those of their affiliated organizations, or those of the publisher, the editors and the reviewers. Any product that may be evaluated in this article, or claim that may be made by its manufacturer, is not guaranteed or endorsed by the publisher.

